# The PGC-1/ERR network and its role in precision oncology

**DOI:** 10.1038/s41698-019-0081-6

**Published:** 2019-03-21

**Authors:** Humberto De Vitto, Ann M. Bode, Zigang Dong

**Affiliations:** The Hormel Institute, University of Minnesota, 801 16th Avenue, Austin, NE 55912 USA

## Abstract

Transcriptional regulators include a superfamily of nuclear proteins referred to as co-activators and co-repressors, both of which are involved in controlling the functions of several nuclear receptors (NRs). The Nuclear Receptor Signaling Atlas (NURSA) has cataloged the composition of NRs, co-regulators, and ligands present in the human cell and their effort has been identified in more than 600 potential molecules. Given the importance of co-regulators in steroid, retinoid, and thyroid hormone signaling networks, hypothesizing that NRs/co-regulators are implicated in a wide range of pathologies are tempting. The co-activators known as peroxisome proliferator-activated receptor gamma co-activator 1 (PGC-1) and their key nuclear partner, the estrogen-related receptor (ERR), are emerging as pivotal transcriptional signatures that regulate an extremely broad repertoire of mitochondrial and metabolic genes, making them very attractive drug targets for cancer. Several studies have provided an increased understanding of the functional and structural biology of nuclear complexes. However, more comprehensive work is needed to create different avenues to explore the therapeutic potential of NRs/co-activators in precision oncology. Here, we discuss the emerging data associated with the structure, function, and molecular biology of the PGC-1/ERR network and address how the concepts evolving from these studies have deepened our understanding of how to develop more effective treatment strategies. We present an overview that underscores new biological insights into PGC-1/ERR to improve cancer outcomes against therapeutic resistance. Finally, we discuss the importance of exploiting new technologies such as single-particle cryo-electron microscopy (cryo-EM) to develop a high-resolution biological structure of PGC-1/ERR, focusing on novel drug discovery for precision oncology.

## Introduction

Transcriptional regulators comprise of nuclear proteins known as co-activators and co-repressors, which bind and control the functions of nuclear receptors (NRs) and transcription factors (TFs).^1–4^ The essential role of NRs and their co-factors in many aspects of mammalian development and physiology raises the possibility that dysfunctions in biological signaling networks controlled by receptors or co-activators, which could be associated with metabolic diseases.^5,6^

Under normal physiological conditions, the proliferator-activated receptor gamma co-activator 1/estrogen-related receptor (PGC-1/ERR) transcriptional axis is involved in the control of mitochondrial biogenesis.^7,8^ Mitochondria are considered to be key regulatory organelles that control cellular survival and death mechanisms, including biomass and energy production for rapid cell growth and apoptosis, respectively.^9^ Hence, many reports support the concept that the PGC-1/ERR pathway plays a dual role in cancer, depending on the specific cellular or tissue context and the environmental stimuli.^10–15^ Notably, the PGC-1/ERR axis has been shown to be essential for functional cancer cell motility and metastasis, leading to malignant transformation in breast and melanoma cancer progression.^14,16,17^ In contrast, this pathway has also been shown to suppress prostate cancer progression and metastasis.^13,18,19^ Although substantial progress has been made in increasing the understanding of the function and molecular biology of NRs and their co-activators, a considerable gap still exists in comprehending how the PGC-1/ERR axis integrates mitochondrial activity through oxidative phosphorylation (OxPhos) leading to cell survival or cell death and how this regulatory function is connected to its dual role in cancer progression.

**Fig. 1:**
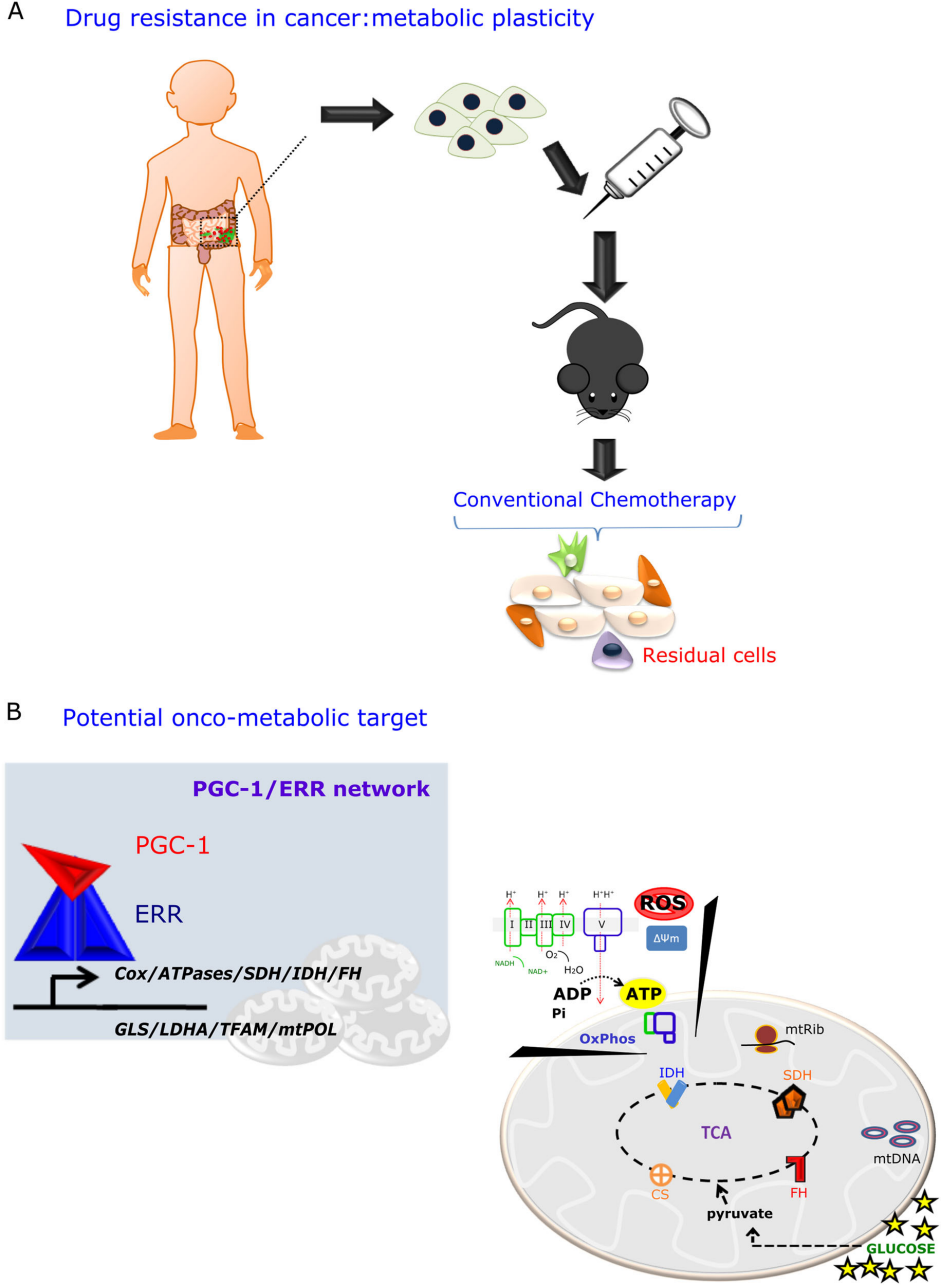
The PGC-1/ERR network as a potential onco-metabolic target in cancer treatment. **a** Cancer translational research methodology based on the patient-derived xenograft (PDX) model highlighting drug resistance in cancer. The survival mechanism of residual cells after conventional chemotherapy relies on metabolic plasticity. **b** The concept of metabolic vulnerability associated with cancer progression can be exploited by targeting a combination of the PGC-1/ERR network and several other mitochondrial weak spots, such as respiratory chain defects, TCA cycle enzymes, including citrate synthase (CS), isocitrate dehydrogenase (IDH), succinate dehydrogenase (SDH) and fumarate hydratase (FH). These enzymes might be exploited as potential onco-metabolic targets, depending on the specific type of cancer

**Fig. 2:**
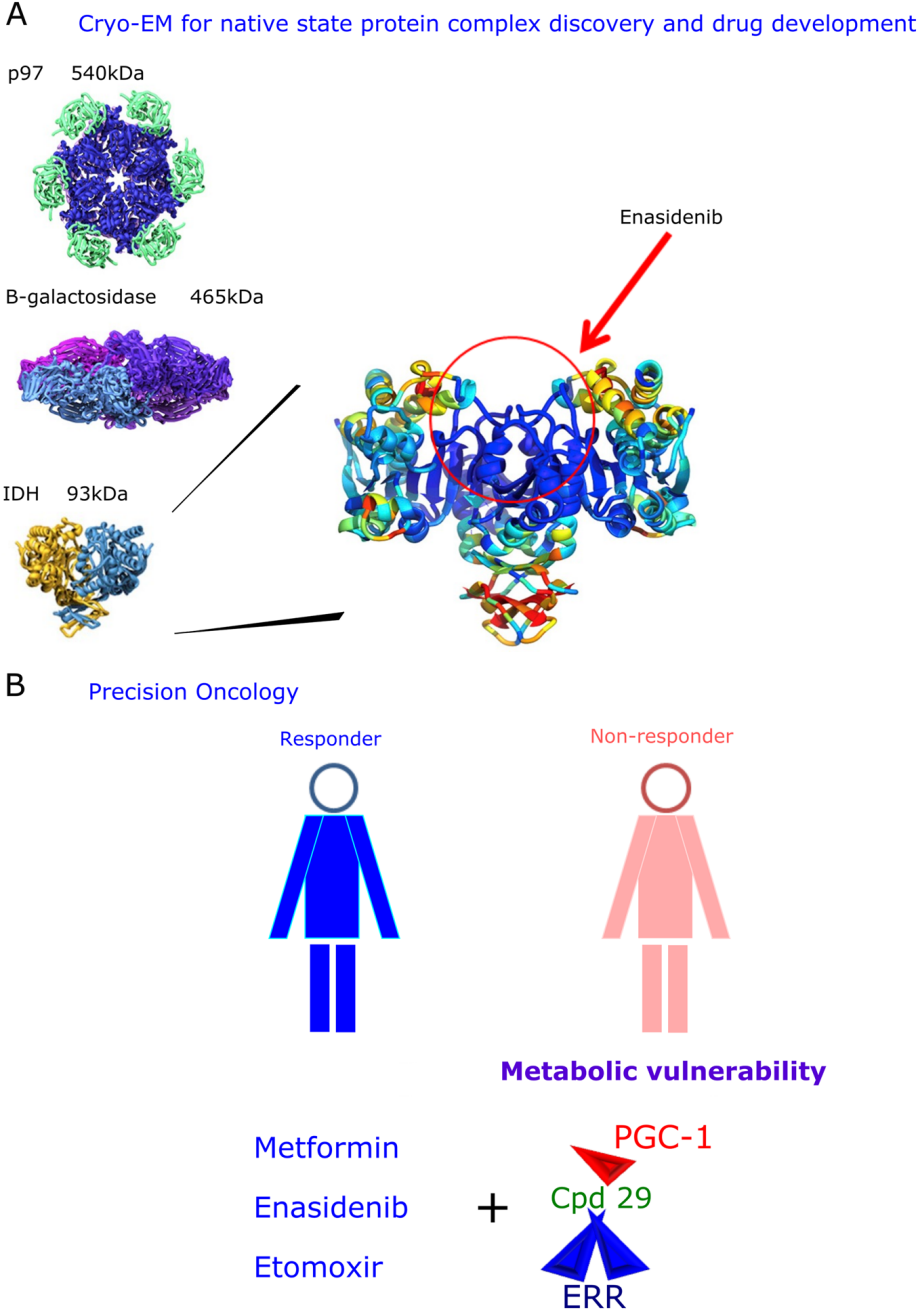
Cryo-EM technology as an important tool for precision oncology. **a** The new cyro-EM technology could provide a better understanding of the native/physiological state of protein complexes for the development of promising therapeutic targets and drug discovery for precision oncology. Reprinted from publication: Merk, A. et al., Breaking Cryo-EM Resolution Barriers to Facilitate Drug Discovery, 1698-707, 2016, with permission from Elsevier.^126^ **b** Mitochondrial inhibitors, like metformin, enasidenib or etomoxir, have been used to treat cancer patients. An ideal scenario to exploit the resistance mechanism in cancer cells that seems to rely on OxPhos activity could be achieved using the concept of metabolic vulnerability, when a combination of targetable genes leads to a lethal phenotype. The PGC-1/ERR axis has great potential to be included as a potential metabolic target for precision oncology for the treatment of non-responder patients. Thus, the combination of mitochondrial inhibitors and inverse agonists of the PGC-1/ERR axis, like compound 29, might provide new hope for treating non-responder patients whose cancer cell survival mechanism relies on mitochondrial metabolism

Additional studies have been conducted that have led to a more comprehensive knowledge of the structure, function, and molecular biology of PGC-1/ERR signaling in cancer biology.^20–24^ Notably, accumulating evidence supports the importance of the PGC-1/ERR transcriptional axis in the context of metabolic-addicted cancer cells.^12,25–29^ This increases the significance of exploiting the therapeutic potential of these targets in an effort to predict the efficacy of therapeutic resistance, the mechanism of which relies on mitochondrial metabolic plasticity (Fig. 1).^12,20,30–32^ This therapeutic potential depends on the function of PGC-1/ERR in coordinating the activity of a broad repertoire of target gene expression associated with mitochondrial biogenesis, OxPhos, energy homeostasis, and glucose, glutamine and lipid metabolism (Table 1).^22,33^ Notably, targeting the PGC-1/ERR network could be accessed by exploring the potential of mitochondrial-linked weak spots, where selective inhibitors of the PGC-1/ERR axis and mitochondrial metabolism might have to be used in combination to target the metabolic addiction of specific cancer cells (Table 1 and Fig. 2).^34^

**Table 1:** Examples of mitochondrial-targeted enzymes regulated by PGC-1/ERR

Metabolic process	Major enzymes	Drug	References
Glycolysis	Hexokinase 2 (HK2)	2-DG, XCT790	^140– 142^
	Lactate dehydrogenases (LDHA, LDHB)	AT-101, FX11, Cpd29	^29, 143^
	Pyruvate kinase (PKM2)	TLN-232/CAP-232	^144, 145^
TCA cycle	Succinate dehydrogenase (SDHB)	3-BrPA, XCT790	^25, 146, 147^
	Isocitrate dehydrogenases−1 and −2 (IDH1, IDH2, IDH3A)	Enasidenib, ivosidenib, Cpd29	^17, 25, 148^
	Fumarate hydratase (FH)	Cpd29	^17, 25^
OxPhos	Mitochondrial complex I	Metformin, phenformin, Cpd29	^11, 17, 149, 150^
Amino acid metabolism	Glutamic-oxaloacetic transaminase−1 and −2 (GOT1, GOT2)	Aminooxyacetate	^25, 151^
Lipid metabolism	Carnitine palmitoyltransferase 1 (CPT1)	Etomoxir	^140, 152^
	Fatty acid synthase (FASN)	Orlistat, cerulenin, TVB-2640	^14, 110^

In an effort to provide new insights into understanding the role of the PGC-1/ERR network, we suggest that new technologies, such as cryo-electron microscopy (cryo-EM), could provide mechanistic comprehension of the biological processes of these protein complexes. The use of cryo-EM could explain the conflicting observations of biochemistry and crystallography that focused only on individual domains or peptides of some of the molecules involved. Moreover, using cryo-EM will allow the investigation of the native state of the PGC-1/ERR complex, which might lead to the identification of novel mechanistic insights into the PGC-1/ERR biology, as well as the discovery of new binding-partners, which could open new therapeutic windows for targeting the PGC-1/ERR complex and its metabolism in precision oncology. For example, Yi et al., used cryo-EM and observed a novel biological insight in the transcriptional activity of the estrogen-receptor (ER) co-activator complex on DNA.^35^ The authors suggested that ER recruits two steroid receptor co-activator 3 proteins and one p300 protein from a DNA-bound complex. The structure of the ER-co-activator complex provided an initial step toward the understanding of the assembly of a full transcriptionally active NR-co-activator complex. Thus, studying the PGC-1/ERR complex in its native state that could shed new light on mechanisms of cancer resistance, which could be better exploited as a therapeutic strategy.

Also, applications of novel technologies that can shed new light on high-resolution biological structures are urgently needed as potential tools to fully elucidate the function and molecular biology of the PGC-1/ERR network in order to be able to further develop promising therapeutic targets and drug discovery for precision oncology (Fig. 2).^36^ Advances in cryo-EM are enabling structure determination of smaller protein complexes without additional modifications such as those required for crystallization that restricts the ability to fully access the mechanistic basis of how cancer metabolism can be orchestrated by these NRs and co-factors.

The main purpose of this review is to provide a critical understanding of the structural biology and function of the PGC-1/ERR network derived from work over the past decade. Moreover, focusing on promising therapeutic targets for precision oncology, this review will explore the underlying potential mechanisms of mitochondrial metabolic targets that could be exploited in combination with the PGC-1/ERR network to improve patient care against therapeutic resistance (Table 1).^30,37^ Understanding this signaling axis could yield crucial insights for the development of novel drugs and therapeutic strategies. This knowledge could lead to a better understanding of the specific type of cancer and patients who are responders and who would benefit from the pharmacological targeting of the PGC-1/ERR network. Thus, the PGC-1/ERR transcriptional axis fits into a novel category of targets that could be useful for exploitation in future research in personalized cancer medicine, so called precision oncology (Fig. 2).

## Structure, function, and molecular biology of PGC-1 and ERR

The PGC-1 co-activator family comprises three different members, PGC-1α, PGC-1β, and PGC-1 related co-activator (PRC). Peroxisome proliferator-activated receptor gamma co-activator 1 alpha (PGC-1α) was first reported to regulate thermogenesis, interacting with the nuclear receptor peroxisome proliferator-activated receptor gamma (PPARγ) in brown adipose tissue (BAT).^38^ The other two members of the PGC-1 family, PGC-1β (PERC) and PRC were described by using sequence homology against PGC-1α.^39,40^

All members of the PGC-1 co-activator family share extensive protein sequence similarity and distinct domains, which could explain their similar physiological role and protein binding-patterns that have already been described.^22^ The human *PPARGC1A* gene on chromosome 4 encodes a 798-amino acid protein also known as PGC-1α. Its homologues, PGC-1β and PRC, comprise 1023 amino acids and 1664 amino acids, respectively. They are encoded by the human *PPARGC1B* gene on chromosome 5 and the human *PPRC1* gene on chromosome 10. Notably, several truncated variants of PGC-1α with distinct transcripts and protein structures have been described. Basically, these variants are generated by alternative splicing and/or differential promoter usage.^41,42^ The presence of different variants of PGC-1α suggests that different protein variants might have distinct transcripts and protein structures with diverse functions, expression levels, and protein–protein interactions, depending on tissue-type or specific disease-context.^41^ More structural and functional studies are needed to address the mechanisms of the regulation of PGC-1α variants and to determine the presence of different variants associated with PGC-1β and PRC.

Structurally, all three members of the PGC-1 co-activator family and all nine variants of PGC-1α have one activation domain localized at their N-terminal region, which contains at least two LXXLL nuclear receptor box motifs.^38,43^ These specific leucine-rich repeats can bind with several NRs, especially the estrogen-related receptor family (ERRα, ERRβ, and ERRγ).^44^ The C-terminal region of all full-length PGC-1s contains a well-conserved RNA-binding domain, including short serine/arginine-rich stretches (RS), a nuclear localization signal (NLS), and the RNA recognition motif (RRM). The RS domain is present only in PGC-1α and PRC, suggesting that both members regulate RNA splicing and processing of mRNAs. However, further investigation is needed for determining whether the RS domain could be involved in processing PGC-1α variants by alternative splicing.^45^ The NLS domain plays a role in the maintenance of PGC-1s inside the nucleus. This domain is missing in alternative PGC-1α variants, which might be found in different cellular compartments, such as the cytosol or mitochondria.^46,47^ The RRM motif also regulates RNA splicing processing of mRNAs, whose function still needs to be substantiated. In addition, other conserved domains have been described in the co-activator family. For example, the aspartic acid (D), histidine (H), aspartic acid (D), and tyrosine (Y) tetrapeptide has been reported to be a binding-partner of the host cell factor (HCF) protein, a transcription factor that regulates gene expression during cell cycle progression. Another motif, including three threonine and four proline amino acids (TPPTTPP), is present in all full-length protein members but the function still needs to be determined.^48^

Functionally, PGC-1α was described as a docking platform for the assembly of transcriptional machinery, forming a macromolecular complex at specific DNA sequences to drive target gene expression.^38^ The same group that first described its activity reported that PGC-1α, even without any histone acetyl transferase activity, promotes gene transcription activity through the formation of a multi-protein complex encompassed by histone acetyltransferase proteins, such as cAMP response element-binding protein-binding protein/p300 and steroid receptor co-activator 1 (SRC-1).^49^ Later, PGC-1α was reported to bind with protein acetyl transferase p300 and the TRAP/mediator complex, mediator of RNA polymerase II transcription subunit 1, leading to the coordination of an important mechanism of chromatin remodeling.^50^ The idea that the interaction between a co-activator and NRs could recruit proteins responsible for chromatin remodeling, histone acetyl transferase activity, and transcriptional activity has emphasized the complex biological network involved in the PGC-1 axis. In vivo studies using double knockout (PGC-1α and PGC-1β) mice suggested that both members of the family share a similar role in the maintenance of mitochondrial function and energetic metabolic demand in many tissues.^51,52^ Conversely, the attempt to generate *PRC*-knockout mice failed because deleting this gene resulted in embryonic lethality.^53^ Nonetheless, in vitro data have shown that PRC plays an important role in mitochondrial biogenesis, but responds to proliferative signals leading to increased cell growth.^54,55^

The molecular biology of PGC-1 has been extensively exploited in different fields of health-related research, including cancer, diabetes, cardiovascular disease, and obesity.^56^ The expression of PGC-1α is characterized by high expression levels in tissues, including kidney, skeletal muscle, liver, heart, neural tissue, and blood mononuclear cells, which exhibit greater energy demand caused by increased mitochondrial activity.^57–59^ The vast number of different tissues or physiological contexts in which PGC-1α is expressed reflects the large number of different NRs and TFs that are regulated by PGC-1α, possibly including all three estrogen-related receptors (ERRα, ERRβ, and ERRγ), SRC-1, and glucocorticoid receptors (GR), as well as the tumor suppressor p53, PPARγ, forkhead box protein O1 (FoxO1), hepatocyte nuclear factor 4α (HNF-4α), nuclear respiratory factor 1 (NRF-1), the cAMP response element binding protein (CREB), and the signal transducer and activator of transcription 6 (STAT-6).^44,60–62^

Most intriguing, the activity of PGC-1α can also be regulated by post-translational modification (PTM) mechanisms.^63^ In fact, most of these regulatory mechanisms are dictated by PTMs, including phosphorylation, acetylation, methylation, ubiquitylation, and O-glycosylation. Clearly, this emphasizes the complex molecular biology and function of the PGC-1 family of proteins. For example, PGC-1α acts as a master regulator of mitochondrial metabolism mediating the entire demand of acetyl-groups and methyl-groups through the tri-carboxylic acid (TCA) cycle and amino acid metabolism, leading to feedback responses of PTM mechanisms, such as acetylation and methylation.^64,65^ Moreover, PGC-1α contributes to ATP production through the OxPhos process to supply the demand of ATP necessary for phosphorylation processes.^66^ Thus, PGC-1α is emerging as a fascinating transcriptional metabolic co-regulator playing a role in the maintenance of a tight equilibrium between metabolic precursors and energy production for sustaining PTM mechanisms.^67^ Besides the complex molecular biology of PGC-1α, more studies need to be conducted to characterize the function of other members of the family.

The molecular biology of PGC-1α is also associated with oncogenes and tumor suppressors. Several studies have demonstrated the interplay between PGC-1α and several oncogenes, including hypoxia-inducible factor 1-alpha (HIF-1α), oncogene carried by the Avian virus, Myelocytomatosis (c-Myc), vascular endothelial growth factor, protein kinase B, and B-Raf proto-oncogene, serine/threonine kinase/microphthalmia-associated transcription factor (MITF), as well as tumor suppressor p53 and 5’ AMP-activated protein kinase (AMPK).^16,62,68–70^ For example, higher PGC-1α expression predicts poor outcome in human melanoma, when the expression of PGC-1α is regulated by the MITF increasing mitochondrial function and resistance to oxidative stress.^16^ Moreover, in wild-type p53 lung adenocarcinoma, PGC-1α binds with p53, promoting cell survival in the presence of metabolic stress.^62,71^ However, the mechanism by which PGC-1α directly modulates oncogenic and tumor suppressor signaling in cancer cells is still unclear.

Different members of the PGC-1 family can act through similar or different molecular mechanisms depending on the cancer type and the stage of disease.^22^ Both PGC-1α and PGC-1β exhibit a similar tissue-specific expression pattern.^44^ As indicated above for PGC-1α, the molecular biology of PGC-1β in cancer is also associated with oncogenes, such as *HIF-1α* and *c-Myc*, and the tumor suppressor gene known as *Von Hippel-Lindau* (*VHL*).^72^ c-Myc is known to control the transcription of the gene encoding PGC-1β^73^ and the VHL/HIF-1 pathway can act as a repressor of c-Myc.^72^ For instance, the loss of PGC-1β and PGC-1α expression is a major factor contributing to impaired mitochondrial respiration in VHL-deficient renal carcinoma cells.^72,74^ Interestingly, the molecular biology of PGC-1β and PGC-1α seems to be modulated toward a metabolic rewiring through the VHL/HIF-1 pathway, leading to “the Warburg effect”, instead of mitochondrial OxPhos metabolism, through the inhibition of the transcriptional c-Myc/PGC-1β axis. Therefore, targeting PGC-1β and PGC-1α and rescuing the mitochondrial metabolic phenotype could be exploited as a therapeutic approach for the treatment of VHL-deficient renal carcinoma cells.^74^ Furthermore, a genomic study designed to assess the activity of ERRα in eight hundred breast tumor samples suggested that the molecular biology of PGC-1β in breast cancer progression relies on the c-Myc pathway. The authors had shown that the insulin-like growth factor 1 receptor pathway controls the stabilization of the c-Myc protein, leading to the up-regulation of PGC-1β.^75^

PGC-1α and PGC-1β were also reported to play a role in the resistance of ER-positive/tamoxifen-sensitive breast tumors.^76^ The interaction between PGC-1β and ERRα mediates a positive transcriptional regulation of receptor tyrosine-protein kinase (ERBB2) expression and co-amplifies genes associated with the ERBB2 amplicon. This biological mechanism was reported as a major factor contributing to tamoxifen resistance in a breast cancer model.^76^ Moreover, PGC-1β mediates adaptive resistance to genotoxic stress in lung cancer associated with mitochondrial DNA mutations.^77^

Taken together, these findings highlight a similar oncogenic network and resistance-related mechanisms in the cancer biology of both members of the PGC-1 family. On the other hand, the third member of the PGC-1 family, called PRC, seems to be restricted to the regulation of the expression of the mitochondrial biogenesis genes in proliferating cells. In contrast to both PGC-1α and PGC-1β, more studies are still needed to determine whether PRC plays a role in cancer progression by associating with oncogenic pathways.^44^ However, one study has recently suggested that the molecular biology of PRC could be associated with the c-Myc pathway in response to mitochondrial stress.^78^

Progress has been made in understanding the signaling network between PGC-1α and ERRα. This network has emerged as an important nuclear transcriptional axis and metabolic signaling pathway in regulating metabolic adaptation in specific cancer types, including breast cancer, prostate cancer, and melanoma.^12^ A majority of the literature has described the function of the founding members of the co-activator family, PGC-1α and ERRα, as a pivotal axis that might be linked to metabolic addiction of specific cancer cells that rely on mitochondrial metabolism for survival.

### ERR

The ERR family encompasses three different members, ERRα (NR3B1), ERRβ (NR3B2), and ERRγ (NR3B3).^79,80^ All three members belong to a subfamily of orphan NRs, sharing sequence homology with the estrogen receptor (ER), but do not require endogenous ligands for activation. In fact, ERRs were first discovered by cDNA library screening using the ERα homology sequence to identify novel steroid receptors.^80^ The DNA-binding domains (DBD) and the ligand-binding domains (LBD) of ERRα and ERα are present in both classical estrogen receptor and orphan receptor families of NRs, but display different molecular biology and function, such as hormone-independent transcriptional activation and ERRα binding with co-activators.^81^ For instance, the ERR-LBD is only 36% similar with the ERs, which could explain why some ERα ligands, such as 17β-estradiol, estrone, and estriol do not activate ERRα.

Structurally, the N-terminal region of ERRs contains a DNA-binding domain and a ligand-independent transcriptional activation function (AF-1) that is poorly conserved within the ERR family members. The N-terminal AF-1 domain can weakly bind several TFs. The recent advances in structural biology have shown that this domain is subjected to various post-translational modifications (e.g., sumoylation and phosphorylation). Only a few groups have described the high-resolution structure of AF-1, which is at least partially due to the very flexible structure and low-affinity nuclear co-activator-binding.^82,83^ The DBD domain can also be acetylated, which controls the affinity by which ERR can bind with its own element responsive sequence (ERRE).^84^ Interestingly, the DBD domain is well-conserved across the members of this subfamily of NRs and contains two highly conserved zinc finger motifs with a specific-DNA binding sequence, TCAAGGTCA. The ERRE is exclusive for the DNA-binding sequence of all three ERR members. However, ERR members have been shown to bind with the responsive element sequence of ER (ERE), suggesting that ERRs play an important role in regulating similar downstream genes controlled by the ER pathway.^82^ The C-terminal region of ERRs comprises an LBD containing a conserved AF-2 helix motif. The LBD of ERR is required for the physical protein interaction with co-activator and co-repressor proteins, such as PGC-1α and PGC-1β or receptor-interacting protein 140 and nuclear receptor co-repressor 1, respectively. The conformation of the LBD of ERR, even in the absence of ligand, is responsible for constitutive transcriptional activation of ERR, due to its unique conformation that facilitates the recruitment of nuclear co-activators.^79^ The LBD domain of ERR binds with the nuclear receptor box motif LXXLL of PGC-1α, forming a binary complex, the stoichiometry of which is defined as two molecules of ERR (dimer) and one molecule of PGC-1α.^21^ Despite the importance of co-repressors in the context of ERR function, we focus herein on the structure, function, and molecular biology of ERR and nuclear co-activators, concentrating on how the PGC-1/ERR network can be exploited as a promising therapeutic target to improve patient outcome. The role of co-repressors and ERRs has been extensively reviewed.^82^

Previously, the observation of a largely occluded ligand-binding pocket in the transcriptionally active conformation of ERRα that has led some to the conclusion that ERRα does not lend itself to direct activation by small molecule agents. However, the crystal structure of the ERRα–LBD has presented an opportunity for generating selective inverse agonists.^85,86^ Currently, a series of diaryl ether-based thioazolidinediones has been screened resulting in the identification of specific inverse agonists of ERRα.^87^ Interestingly, compound 29 was obtained for the ERRα–LBD by solving the X-ray crystal structure.^85^ Compound 29 acts as a ligand of ERRα through a covalent interaction leading to conformational changes (in the amino acid Phe328) that disrupts the interaction between ERRα and PGC-1α.^87^ The biological consequence of this binding has shown growth-inhibitory therapeutic effects in certain cancers such as breast cancer and melanoma.^17,29,88^ Furthermore, several other compounds have been developed as inverse agonists of ERRα, such as compound 1a, compound 3, N-arylindole, XCT790, and GSK0903. However, more biological study is needed to determine the specificity of these compounds in targeting only the PGC-1/ERR network. For instance, XCT790 was previously developed as a specific inverse agonist of ERRα with the capacity to disrupt the interaction between ERRα and PGC-1α,^89^ leading to growth-inhibitory therapeutic effects in breast cancer.^29^ However, XCT790 does not seem to be a very specific inverse agonist of ERRα because at nanomolar concentrations (10-fold lower than the concentration required to inhibit ERRα), XCT790 is a potent mitochondrial uncoupler, leading to a rapid depletion of ATP and activation of AMPK. The authors suggested that XCT790 is a potent, fast-acting, mitochondrial uncoupler that acts independent of its inhibition of ERRα.^90^

Notably, Kallen and co-workers showed for the first time the X-ray crystal structure of the ERRα-LBD with one co-activator peptide derived from PGC-1α, leading to a ligand-independent transcriptional activation by ERR.^91^ In 2007, the same group used an inverse agonist called compound 1a to show that the binding interface of this compound with the ERRα-LBD comprised the helix H12 together with helices H3 and H4. Interestingly, the compound imposed dramatic conformational changes in the amino acid Phe328 located at H3, moving away the amino acid Phe510 of H12, which contains the co-activator groove of the ERRα-LBD. Based on this evidence, they proposed a novel molecular mechanism supporting the idea that the helix H12 binds with the co-activator peptide or compound 1a filling the co-activator groove of the AF-2 domain of ERRα. Hence, no co-activators and co-repressors are allowed to interact with ERRα.^85^ The X-ray crystal structure of the ERRα-LBD with compound 29 displayed similar dramatic conformational changes and side chain rotation of the amino acid Phe328 (H3). The significant change in structure was observed in the loop between helices H11 and H12. Hence, the C-terminal AF-2 domain that is responsible for transducing the constitutive activity for ERRα is no longer functional.^87^

As indicated earlier, the function and molecular biology of ERRs under normal physiological conditions are associated with the regulation of metabolic genes that are involved in glycolysis, the TCA cycle, and mitochondrial metabolism. These receptors also influence enzymes participating in OxPhos (e.g., several components of mitochondrial respiratory complexes), amino acid metabolism, and lipid synthesis (Table 1).

However, in the context of cancer, the different members of the ERR family seem to exhibit distinct functions in cancer progression. ERRα expression is associated with poor prognosis in breast tumors because it appears to drive lapatinib-resistance and tamoxifen-resistance in those patients.^27,92^ Several studies have shown that ERRα expression is associated with an increased risk of recurrence and worse prognosis, as well as drug resistance in patients with breast cancer.^92–94^ ERRα has been shown to increase the expression of ERBB2, mediating endocrine-resistant ERα-positive cells.^76,95^ Interestingly, ERRα has been shown to mediate pro-survival functions and represents a novel therapeutic target in a particularly aggressive melanoma phenotype, known as PGC-1α-positive melanomas.^17^

In contrast, the role of the ERRγ isoform in cancer biology seems to be paradoxical, whether this NR functions as an oncogene or as a tumor suppressor. Recently, genomic analysis in gastric cancer revealed that ERRγ acts as a tumor suppressor by directly targeting the Wnt signaling pathway. In fact, activating ERRγ expression by a specific agonist, DY131, inhibits gastric cancer cell growth and improved patient prognosis.^96^ Furthermore, ERRγ was described as an anti-proliferative target in androgen-sensitive and androgen-insensitive prostate cancer cells.^97^ Conversely, ERRγ is up-regulated in liver cancer and its inhibition suppresses cancer cell survival through the p21 and p27 proteins.^98^

Most intriguing is the paradoxical function of ERRγ in breast cancer progression. Recently, several studies reported that the hyper-activation of ERRγ induces a pro-survival transcriptional program in tamoxifen-treated breast cancer, as reported to the isoform ERRα.^99,100^ Conversely, in breast tumors co-expressing ER and PR, ERRγ induces E-cadherin expression and promotes the mesenchymal-to-epithelial transition (MET), resulting in the inhibition of tumor growth.^101,102^ In spite of the inconsistencies, the expression of ERRα and ERRγ in breast cancer and prostate cancer seems to be inversely correlated, where the ERRα is associated with a more aggressive disease and the expression of ERRγ is associated with a favorable prognosis of patients with breast and skin cancer.^81,103^ For instance, in androgen-dependent and castration-resistant prostate cancer, the progression of the disease is associated with a loss of ERRγ expression, whereas strategies to reactivate ERRγ expression could be exploited as a generalized therapeutic approach to manage prostate cancer.^19^

Overall, ERRα and ERRγ are considered key regulators of metabolic reprogramming in breast and prostate cancer. However, how this nuclear receptors network influences the metabolic state of cancer cells seems to be very complex and diverse, depending on the cancer type. Regarding the third isoform of this family, in prostate cancer the expression of ERRβ transactivates a promoter upstream of the cyclin-dependent kinase inhibitor, p21 gene, resulting in the inhibition of cell cycle progression, whereas the potential role of ERRβ in breast cancer remains unclear.^104^

Clearly a close relationship exists between PGC-1/ERR activity and cancer therapeutic resistance. New insights into the PGC-1α/ERRα network in cancer will be discussed next.

## New biological insights into the PGC-1α co-activator and ERRα

Reprogramming of energy metabolism and evading immune destruction have been recently included in the select list of biological capabilities or hallmarks acquired during the development of cancer.^105^ Notably, the plasticity of cancer cells toward metabolic reprogramming has gained attention in the mechanisms of drug resistance.^30,106–108^ The entire molecular network that orchestrates the inherent ability of tumor cells to switch between different metabolic profiles, depending on the microenvironment stimuli, still needs to be fully elucidated.

The most important metabolic plasticity mechanisms in cancer rely on glycolysis-dependent or mitochondrial OxPhos-addiction activities.^109,110^ Both are considered metabolic hallmarks of cancer cells because they are involved in the direct activation of many oncogenic pathways.^107,111–113^ However, not all reprogrammed metabolic activities contribute equally to cancer progression. Yet, deregulation of mitochondrial metabolism could be considered a potential therapeutic target in tumor resistance.^114^ Because the PGC-1α/ERRα network is a master regulator of mitochondrial biogenesis, it could be considered a nodal regulatory step capable of controlling the entire cellular metabolism and, at least in part, in modulating this resistance-related mechanism in cancer, leading to cancer recurrence (Fig. 1).^115,116^

Recent studies have provided a rationale for therapeutically targeting mitochondria in certain types of cancer.^11,16,117,118^ Different modulators of mitochondrial activity that have been approved by The Food and Drug Administration (FDA) to treat chronic diseases, such as type 2 diabetes, cardiovascular disease, obesity, and acute myelogenous leukemia (AML), are currently being explored in clinical trials to determine potential efficacy to treat cancer. For example, anti-diabetic drugs like metformin and phenformin, the anti-obesity drug, etomoxir, or the anti-AML drug, enasidenib (IDHIFA), might provide new hope for treating responder patients whose cancer cell survival mechanism relies on mitochondrial metabolism.^119^ Although the clinical use of these drugs in combination with conventional chemotherapeutic drugs has led to clinical improvement outcomes, the drug-resistance mechanisms of cancer cells still remain unclear.

### From metabolism to precision oncology

To focus on precision oncology and the development of effective drug design to eradicate drug-resistant cancer cells, the signaling network associated with PGC-1α/ERRα must be considered a novel targetable vulnerability in cancer cells. In examining the concept of metabolic rewiring, targeting this particular transcriptional/mitochondrial metabolic network might expose other vulnerabilities to oxidative stress in tumors (Table 1).^34^ Thus, targeting PGC-1α/ERRα in combination with additional metabolic vulnerabilities such as the respiratory chain defects, antioxidant programs, and TCA cycle enzymes might lead to the disruption of the nutrient sensing pathways responsible for survival of residual cells (Fig. 2b).^120^

A thorough analysis of the literature shows that many of the human NRs and their co-activators have been extensively studied using traditional structural analysis. However, only studies with partial protein structure and limited protein–protein interaction have been purposed, which has led to an incomplete understanding of the entire functional mechanism of the PGC-1/ERR network.^21,85,91,121,122^ Therefore, new insights into the overall structure of the PGC-1/ERR complex could provide insights for effectively targeting cancer resistance mechanisms and answering important questions associated with the function of this nodal transcriptional signaling network. Essential questions that could be answered include whether the full-length protein structure of both components would impose particular changes in the protein–protein interaction model that could affect the discovery and development of new therapies targeting this complex. In addition, structural insights could assist in determining whether the PGC-1α and ERRα proteins interact with specific oncogenes or tumor suppressors and thus play different roles in metabolic plasticity favoring drug resistance or decreasing cancer progression.

In this context, recent advances have been made in successfully determining high-resolution biological structures. Solving the PGC-1/ERR complex structure in its physiological state might lead to the discovery of novel mechanistic insights into the biology of the PGC-1/ERR axis, as well as the identification of novel binding partners that might have clinical relevance to treat cancers that rely on mitochondrial activity.^17,123^ Therefore, more studies must be pursued to predict how this complex might be exploited in basic and clinical research, leading to the elucidation of dynamic biological processes in their native states and drug discovery for personalized medicine.

### Cryo-EM as a potential tool for the visualization of protein complexes

Cryo-EM is an outstanding new technology that is based on transmission electron microscopy in which a protein sample is examined in its native state at cryogenic temperatures, which can lead to successful resolution of the protein’s structure at the subatomic or atomic level. This technology has several advantages over X-ray crystallography because the protein is frozen in its native state, which can overcome technical problems with proteins that are refractory to crystallization or are just difficult to crystallize. Cryo-EM has been used successfully to resolve proteins of greater than 300 kDa to produce images with resolution as good as 2.2 Å. For example, the large β-galactosidase protein (465 kDa) has been successfully resolved by cryo-EM.^124^ Notably, high resolution cryo-EM images could reveal protein–protein interactions, conformational changes, and interactions between proteins and drug targets at an atomic level of precision.^125^ Despite the lower size limitation of protein structures that can be resolved by cryo-EM, one study showed that cryo-EM is suitable to solve the structure of small metabolic enzymes at near-atomic resolution.^126^ The authors presented the structure of a known therapeutic cancer target, isocitrate dehydrogenase (IDH, 93 kDa), with a resolution of 3.8 Å, which could facilitate research toward therapeutic targets and drug discovery. This is possible because crossing the 3 Å resolution level and obtaining protein structures with sizes <100 kDa might allow scientists to investigate drug-target interactions and dynamic conformational states of protein complexes (Fig. 2a).^126,127^ This could even benefit patients already treated with conventional chemotherapies, such as lapatinib and tamoxifen in metastatic breast cancer, epidermal growth factor inhibitors (EGFRi) in EGFR-driven lung adenocarcinoma, mitogen-activated protein kinase inhibitors (MAPKi) in melanoma, and 5-fluorouracil in Myc/PGC-1α-driven pancreatic cancer.^30^

## The PGC-1/ERR network as a promising therapeutic target for precision oncology

Panomics data, including genomics, transcriptomics, proteomics and metabolomics, in combination with patient-matched data, are currently being used for designing treatments for personalized medicine.^128^ New technologies, including the clustered regularly interspaced short palindromic repeats-associated 9 system for targeted genome editing and cryo-EM, can provide mechanistic understanding of complex biological processes and are the potential tools for identifying promising therapeutic targets for precision oncology.^36^

The relevance of deciphering the role of the PGC-1/ERR signaling network and the therapeutic implication for precision oncology relies on several factors. First, the PGC-1/ERR transcriptional network is responsible for metabolic plasticity, which corresponds well with therapeutic resistance.^42^ For example, like all cancers, breast cancer is considered a heterogeneous disease and currently the approach to treat breast cancer is still based on histopathological markers that rely on tumor subtypes to evaluate and treat each patient.^129–131^ In this era of precision oncology, seeking clinically relevant biomarkers that might be exploited for therapeutic purposes is highly pertinent and could be combined with conventional therapies to generate synthetic lethality in breast cancer.^132,133^ Synthetic lethality is defined as any genetic mutation, chemical or drug perturbation, and environmental conditions that have a unique effect on cell viability but when exploited in combination results in cell death.^134^ For instance, the concept of synthetic lethality is used to treat breast cancer based on the treatment of BRCA1-deficient patients with PARP inhibitors.^135^ However, the means by which the PGC-1/ERR axis can be targeted to interfere with the metabolic synthetic lethality of mitochondrial enzymes still remains largely unknown.^34^ Second, the PGC-1/ERR complex is an important nuclear transcriptional axis that orchestrates the mitochondrial bioenergetic requirements of tumors and thereby it could be therapeutically exploited in metabolic-addictive cancers as a new metabolic vulnerability.^136^ Third, preventing or bypassing drug resistance is arguably the most important medical need in cancer research.^114^ Cleary, the identification of biomarker-defined patient populations that will most likely respond to specific drugs is critical.^114^ Thus, silencing the PGC-1/ERR axis in drug-resistant cancers with a high level of OxPhos might culminate in specific elimination of these cells. Finally, the attempt to access the native and physiological state of PGC-1/ERR axis will allow us to a greater understanding of the PGC-1/ERR axis in cancer biology. Moreover, the potential discovery of important oncogenes or tumor suppressors that interact with this complex will lead to the ability to fully access the mechanistic basis of how these NRs and co-activators orchestrate metabolic plasticity toward drug-resistance in cancer treatment.

## Concluding remarks

Despite the progress that has been made in using structural biology for potential drug discovery to increase patient outcomes underlying precision oncology, new therapies that effectively eradicate drug-resistant cancer cells are an immediate clinical necessity. The PGC-1/ERR network holds promise as a therapeutic target for precision medicine, because this transcriptional axis orchestrates the expression of several genes involved in mitochondrial biogenesis and cell metabolism. Bosc et al. have suggested that the resistance mechanism in cancer might be associated with a shift toward an increased OxPhos status that should be considered a distinctive characteristic of drug resistance.^30^ The master PGC-1/ERR axis that controls mitochondrial OxPhos activity should be considered as a new pathway that drives resistance in tumor progression. The metabolic vulnerability concept that explores potential mitochondrial targets to treat cancer, rather than conventional chemotherapy, extends the concept that the up-stream mitochondrial biogenesis PGC-1/ERR network must be included as a novel targetable metabolic vulnerability (Fig. 2b). Furthermore, a native-state high-resolution structure of this nuclear complex is urgently needed.^89,137–139^ Overall, further studies are needed to determine the role of PGC-1/ERR network as a key metabolic vulnerability associated with cancer cell progression by using cryo-EM as a promising tool for drug discovery in precision oncology. Such refinements could provide opportunities to be exploited in therapeutic resistance.

### Reporting summary

Further information on experimental design is available in the Nature Research Reporting Summary linked to this article.

## Supplementary information

Reporting Summary

## Data Availability

Data sharing not applicable to this article as no datasets were generated or analyzed for the current study.

## Supplementary information

**Supplementary information** accompanies the paper on the *npj Precision Oncology* website (10.1038/s41698-019-0081-6).
